# The Herb Medicine Formula “Chong Lou Fu Fang” Increases the Cytotoxicity of Chemotherapeutic Agents and Down-Regulates the Expression of Chemotherapeutic Agent Resistance-Related Genes in Human Gastric Cancer Cells *In Vitro*

**DOI:** 10.1093/ecam/nep175

**Published:** 2011-06-05

**Authors:** Yongping Liu, Yang Ling, Wenjing Hu, Li Xie, Lixia Yu, Xiaoping Qian, Binxia Zhang, Baorui Liu

**Affiliations:** ^1^Department of Oncology, Nanjing Drum Tower Hospital, Nanjing University of Traditional Chinese Medicine, Zhongshan Road 321, Nanjing 210008, China; ^2^Clinical Oncology Laboratory, Changzhou Tumor Hospital Affiliated to Medical School of Suzhou University, Huaide Road 44, Changzhou 213002, China; ^3^Department of Internal Medicine, Traditional Chinese Medicine Hospital of Changzhou, Heping Road 156, Changzhou 213002, China

## Abstract

The herb medicine formula “Chong Lou Fu Fang” (CLFF) has efficacy in inhibiting the proliferation of human gastric cancer *in vitro* and *in vivo*. To explore the potentially useful combination of CLFF with chemotherapeutic agents commonly used in gastric cancer therapy, we assess the interaction between CLFF and these chemotherapeutic agents in both SGC-7901 cell lines and BGC-823 cell lines using a median effect analysis and apoptosis analysis, and we also investigate the influence of CLFF on chemotherapeutic agent-associated gene expression. The synergistic analysis indicated that CLFF had a synergistic effect on the cytotoxicity of 5-fluorouracil (5-FU) in a relative broad dose inhibition range (20–95% fraction affected in SGC-7901cell lines and 5–65% fraction affected in BGC-823 cell lines), while the synergistic interaction between CLFF and oxaliplatin or docetaxel only existed in a low dose inhibition range (≤50% fraction affected in both cell lines). Combination of CLFF and chemotherapeutic agents could also induce apoptosis in a synergistic manner. After 24 h, CLFF alone or CLFF combination with chemotherapeutic agents could significantly suppress the levels of expression of chemotherapeutic agent resistance related genes in gastric cancer cells. Our findings indicate that there are useful synergistic interactions between CLFF and chemotherapeutic agents in gastric cancer cells, and the possible mechanisms might be partially due to the down-regulation of chemotherapeutic agent resistance related genes and the synergistic apoptotic effect.

## 1. Introduction

Despite its declining incidence, gastric cancer is still the second most common cause of death from cancer in Asia and worldwide [1, 2]. Surgery remains the mainstay of any curative treatment; however, approximately two-thirds of patients diagnosed with gastric cancer have unresectable locally advanced and/or metastatic disease [3]. Most patients with advanced gastric cancer need to accept cytotoxic chemotherapy as one of main treatments. Recently, platinum compounds, 5-fluorouracil and taxanes have been widely used in treatment of gastric cancer. Various attempts have been made to improve the objective response rate to chemotherapy, including chemotherapeutic agents in combination, however, the optimal combination regimen has remained elusive, and standard treatment remains a matter of debate [4]. It is necessary to find new compounds and optimized combinational treatment for gastric cancer.

Herb medicine as complementary and alternative therapy (CAM) has enjoyed a growing popularity in cancer patients as a less intensive and more natural approach to alleviating the side effects of chemotherapy or improving quality of life [5–7]. And more and more cancer patients select herb medicine as CAM in combination with their conventional chemotherapeutic treatment [8], which increases the probability of clinically relevant herb-chemotherapeutic agents interactions. Considering the narrow therapeutic window of chemotherapeutic agents, the synergistic or additive interactions may increase the outcomes of the therapy and decrease the dosages of chemotherapeutic agents. CLFF is a prescribed complex of Chinese herbal formula containing three ingredients as follows: *Rhizoma Paridis*, *Fructus Forsythiae* and *Radix Codonopsis*, in which *R. Paridis* represents the principal ingredient, while *F. Forsythiae* and *R. Codonopsis* serve as adjuvant ones to assist the effects of *R. Paridis*. In traditional Chinese medicine (TCM), *R. Paridis* as principal component of many herbal formulae has been widely used as CAM in various kinds of cancers, such as gastric cancer, lung cancer, hepatic carcinoma, cervical cancer and so on [9]. Because of different adjuvant components, every formula has a different name or no name, and precise mechanisms of these formulae also remain to be addressed by using molecular approaches. CLFF as one of those formulae is being studied in our laboratory, and two components of CLFF, *R. Paridis* and *F. Forsythiae* have been proved to have potential anticancer activity on digestive cancer in our preliminary experiment [10, 11], and *R. Codonopsis*, another component of CLFF, has also long been used for replenishing energy deficiency, strengthening the immune system, lowering blood pressure and improving appetite in China [12]. *In vitro* and *in vivo* studies have indicated that CLFF has prominent cytotoxic effect and potential immune-modulating function on many kinds of gastrointestinal cancers, but not has significant side effects (another parallel study). However, the potential role of this formula in cancer therapy has not been clearly addressed by modern science.

This preclinical study was therefore undertaken to investigate whether combination of CLFF and a series of chemotherapeutic agents could result in a useful synergistic interaction against gastric cancer cells. To elucidate further the mechanisms possibly involved in the interaction between the CLFF and chemotherapeutic agents, we also investigated apoptosis and expression of chemotherapeutic agent resistance related genes in gastric cancer cells after treatment with CLFF and chemotherapeutic agents singly and in combination.

## 2. Methods

### 2.1. Preparation of CLFF Extract

Medicinal plants were provided by Nanjing herb Pharmaceutics Ltd. (Nanjing, China) for the preparation of the CLFF extract. The preparation is a mixture of three crude plant extract ingredients: *R. Paridis*, *F. Forsythiae* and *R. Codonopsis*, at a ratio of 10 : 5 : 5. The plants were homogenized with a Waring blender, then soaked in 10 l (10-fold of the plant) double distilled water (DDW) for 24 h. The mixture was heated to 100°C for 1 h, and the decoction was filtrated. The filtrates obtained from three cycles of the procedures were mixed, concentrated by heating and granulated by lyophilization. Total yield of the CLFF extract is 125 g lyophilized powder from water extract of 1 kg raw mixed herb. Aqueous solution was prepared by dissolving the granulated formulae in water at 250 mg raw mixed herb/ml for CLFF and filtered through a 0.2 mm filter (Microgen, Laguna Hills, CA, USA) before use. The quality control on CLFF preparation, including definition of the correct plants, origin of production, implantation, harvesting and processing, was according to the guidelines defined by Chinese State Food and Drug Administration (SFDA). The species, plant parts and origin used in the formula as Table 1.

### 2.2. Cell Lines and Cell Culture

Human poorly differentiated SGC-7901 cells was kindly provided by the center laboratory of the Second Hospital, Chang Zhou (China), BGC-823 cells was obtained from Shanghai Institute of Cell Biology (Shanghai, China). All cell lines were propagated in RPMI 1640 medium (GIBCO BRL), supplemented with 10% bovine serum, penicillin (100 U ml^−1^)—streptomycin (100 *μ*g ml^−1^), pyruvate, glutamine and insulin at 37°C in a water-saturated atmosphere with 5% CO_2_.

### 2.3. Drugs

5-Fluorouracil (5-FU), oxaliplatin (Oxa) and docetaxel (Doc) were supplied from Jiangsu Hengrui Medicine Company (Jiangsu, China). The dilutions of all of the reagents were freshly prepared before each experiment. CellTiter 96 AQueous One Solution Cell Proliferation Assay kit was purchased from Promega (Madison, WI, USA). Annexin-V-FITC Apoptosis Detection Kit was purchased from Invitrogen (Carlsbad, CA, USA).

### 2.4. Cytotoxicity Assay and Analysis of Combination Effects

Cytotoxicity was determined by CellTiter 96 AQueous One Solution Cell Proliferation Assay kit. Briefly, Tumor cells growing in log-phase were trypsinized and seed at 2 × 10^3^ cells per well into 96-well plates and allowed to attach overnight. Medium in each well was replaced with fresh medium or medium with various concentrations of drug in at least six replicate wells and left contact for 48 h. One–fifth volume of CellTiter 96 AQueous One Solution was added to each well and incubated for an additional 3 h, Absorbance was determined with a microplate reader (BIO-RAD) at 490 nm. The blank control wells were used for zeroing absorbance. Each experiment was allocated 10-wells containing drug-free medium for the control. The inhibition rate (*I*%) was calculated using the background-corrected absorbance by the following equation: *I*% = 100 × (*A*_untreated  control  well_ − *A*_experimental  well_)/*A*_untreated  control  well_. The IC50 was defined as the concentration required for 50% inhibition of cell growth.

For the combination experiments, drugs were added either concomitantly or sequentially with seven different concentrations of the single agent and seven different concentrations of both agents at their fixed concentration ratio for 48 h. The fractional inhibition of cell proliferation was calculated by comparison to control cultures. Dose-response curves were obtained for each drugs, and for multiple dilutions of a fixed-ratio combination of the two drugs. Median effect analysis using the combination index (CI) method of Chou and Talalay [13] was employed to determine the nature of the interaction observed between CLFF and chemotherapeutic agents. The CI is defined by the following equation: CI = (*D*)*_1_/*(*Dx*)*_1_ +* (*D*)*_2_/*(*Dx*)*_2_ + *α**(*D*)*_1_*(*D*)*_2_/*(*Dx*)*_1_*(*Dx*)_2_, in which (*Dx*)_1_ and (*Dx*)_2_ are the concentrations for *D*_1_ (CLFF) and *D*_2_ (chemotherapeutic agent) alone that gives *x*% inhibition, whereas (*D*)_1_ and (*D*)_2_ in the numerators are the concentrations of CLFF and another drug that produce the identical level of effect in combination. *α* = 0 when the drugs are mutually exclusive (i.e., with similar modes of action), while *α* = 1 when they are mutually non-exclusive (i.e., with independent modes of action). CIs > 1 indicate antagonism, CIs < 1 indicate synergy and CIs = 1 indicate additivity. Each CI ratio represented here is the mean value derived from at least three independent experiments.

### 2.5. Apoptosis Assay

Cells quantification of apoptosis cells was performed using an Annexin-V-FITC Apoptosis Detection Kit (Invitrogen, Carlsbad, CA, USA) according to the manufacturer's instructions. Briefly, cells were plated in a 60-mm Petri disk and allowed to grow to 75–80% confluence. They were exposed to CLFF and anticancer drugs added singly or in combination for 48 h and compared with control cells not treated with drugs. Then cells were collected and resuspended in 500 *μ*l of binding buffer, and 5 *μ*l of Annexin-V-fluorescein isothiocyanate (FITC) and 5 *μ*l of propidium iodide (PI) were added. Analyses were performed with a flow cytometer (FACScalibur, Becton Dickinson, Franklin Lakes, NJ, USA).

### 2.6. Quantitative RT-PCR

Cells were harvested with trypsin, washed with PBS, and collected by centrifugation at 1000 rpm for 5 min. Total RNA was extracted using SV Total RNA isolation system (Promega, Madison, WI, USA) following the manufacturer's protocol. And its purity and quality were measured by Bio-visible spectrophotometer (Eppendorf, Germany); 1% agarose gel electrophoresis was used to assess the integrity of the obtained RNA. cDNA with a total volume of 20 *μ*l was synthesized using the reverse transcription system containing reverse transcriptase (Promega, Madison, WI, USA) according to the recommended protocol by the manufacturer. Real-time quantitative PCR of the target gene and *β*-actin as internal control was carried out with icycler iQ Multicolor Real-time PCR Detection System (Bio-Rad Laboratories, Inc.). The 20 *μ*l PCR reaction mixture contained 1× primers and probe mixture (Applied Biosystems, Foster city, CA. Assay IDs: Hs00157415_m1 (ERCC1); Hs00426591_m1 (TS); Hs00964965_m1 (*β*-tubulin III); Hs00213491_m1 (tau); Hs99999903_m1 (*β*-actin)), 1× Absolute QPCR Mix (ABgene, Surrey, UK). The PCR conditions were 50°C for 2 min, 95°C for 15 min, followed by 45 cycles at 95°C for 15 s and 60°C for 1 min. Relative gene expression quantifications were calculated according to the comparative *C*_T_ method using *β*-actin as an endogenous control and commercial human total RNA (BD Clontech, CA, USA) as calibrators. Final results were determined by the formula2^−ΔΔ^ *^C^* ^T^ method [14].

### 2.7. Statistical Analysis

Values were expressed as means ± standard deviations. Statistical comparison was performed using Student's *t*-test, and a *P*-value of <.05 was considered statistically significant.

## 3. Results

### 3.1. Cytotoxicities of CLFF and Chemotherapeutic Agents against SGC-7901 and BGC-823 Cells

The cytotoxic activities of CLFF and chemotherapeutic agents were tested individually on both two cell lines. As expected, CLFF and each chemotherapeutic agent individually increased the cytotoxicity of both two cell lines in a dose-dependent fashion.  Table 2 shows the IC50 doses for both SGC-7901 and BGC-823 cells lines following exposure to CLFF or chemotherapeutic agents. The response of both two cells lines to these drugs was significantly different (*P* < .05). And it appeared that SGC-7901 cells was more sensitive to CLFF, 5-FU and Doc, while BGC-823 cells was more sensitive to Oxa, suggesting the genetic make-up of the cells plays an important role in the response to drug treatment.

### 3.2. Median-Effect Analysis of CLFF and Chemotherapeutic Agent Combination In Vitro

**Figure 1:**
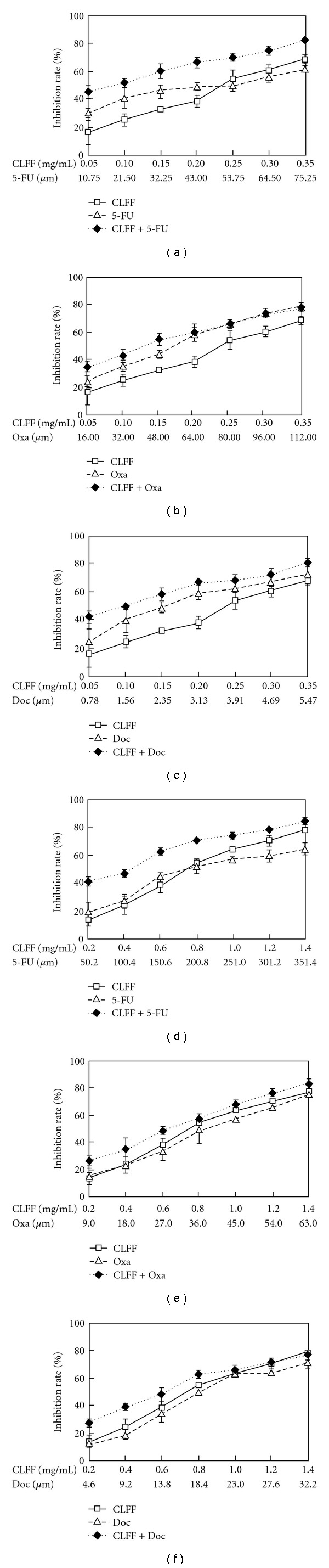
Dose-response curve of CLFF and chemotherapeutic agents singly or in combination for SGC-7901 (a–c) and BGC-823 (d–f) cells. Data point, means of at least three independent experiment; bars, SD, standard deviation.

**Figure 2:**
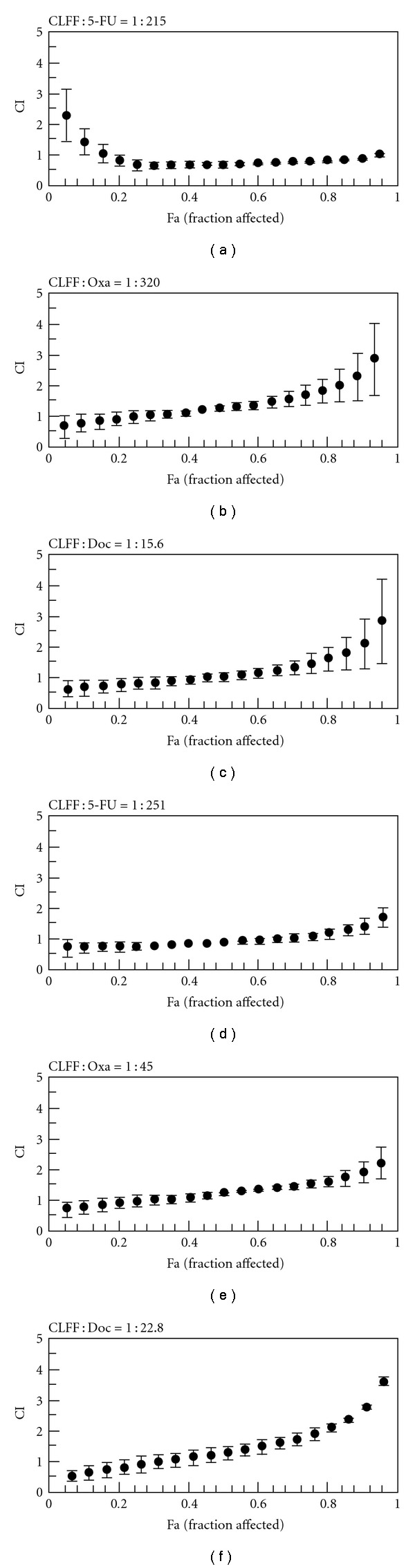
Combination index values for simultaneous treatment of CLFF and chemotherapeutic agents in SGC-7901 (a–c) and BGC-823 (d–f) cells. Cells were simultaneously exposed to two agents at fixed ratios for 48 h at 37°C in 5% CO_2_. Data point, means of at least three independent experiment; bars, SD; (a, d) CLFF plus 5-fluorouracil; (b, e) CLFF plus oxaliplatin; (c, f) CLFF plus docetaxel. CI < 1, =1 and >1 indicate synergism, addition and antagonism, respectively.

**Figure 3:**
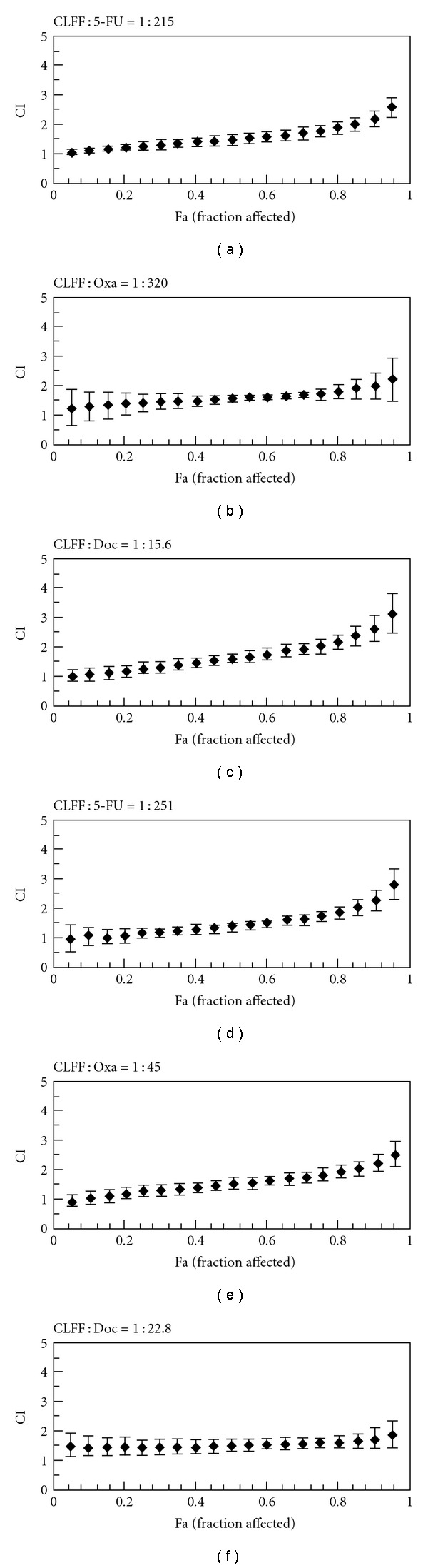
Combination index values with the treatment schedule chemotherapeutic agents preceding CLFF in SGC-7901 (a–c) and BGC-823 (d–f) cells. Cells were pretreated with every chemotherapeutic agent for 24 h, followed by CLFF at fixed ratios for 48 h at 37°C in 5% CO_2_. Data point, means of at least three independent experiment; bars, SD; (a, d) CLFF plus 5-fluorouracil; (b, e) CLFF plus oxaliplatin; (c, f) CLFF plus docetaxel. CI < 1, =1 and >1 indicate synergism, addition and antagonism, respectively.

To explore whether CLFF could enhance the effects of the chemotherapeutic agents currently used to treat gastric cancer, the effects of 48 h treatment with CLFF, 5-FU, Oxa and Doc singly and in combination were examined.  Figure 1 shows the dose-response curves for SGC-7901 and BGC-823 cells lines exposed to CLFF and chemotherapeutic agents singly and in combination. For both cell lines, drug combinations gave a more intensive inhibition of cell proliferation. Especially combination of 5-FU and CLFF resulted in more effective inhibition of cell proliferation in almost all range of inhibition rates. To fully evaluate the nature of the interaction between CLFF and chemotherapeutic agents, we analyzed the combination of both drugs using media-effect analysis, which resolves the degree of synergy, addictively, or antagonism at various levels of cell death.  Figure 2 illustrates the multiple drug effect obtained for SGC-7901 and BGC-823 cells, which were treated simultaneously with CLFF and anticancer drugs and represented as fractional cell growth inhibition (FA) as a function of the CI. As shown in Figure 2, when cells were treated with CLFF and 5-FU simultaneously, the CI values were below 1 in a relative broad range of killed cell fraction in both cell lines, which indicated that CLFF had a synergistic effect on the cytotoxicity of 5-FU in a more broad range of dose inhibition rate. While combination of CLFF and other two chemotherapeutic agents resulted in synergistic effects only at lower levels of killed cell fraction in both cell lines (<50% killed cell fraction). The results are summarized in Table 3, which shows, for each combination, the computer-calculated CI for 20, 30, 50 and 80% cytotoxicity (Fa = 0.2, 0.3,0.5, 0.8, resp.). It was different in some extent of concentrations between two cell lines. But in all combinations, the CI values were below 1 at Fa = 0.3, indicating a synergistic anti-proliferative effect at lower levels of killed cell fraction. We also evaluated the effects of sequential drug exposure, in which either CLFF or chemotherapeutic agents were administered alone for 24 h before administration of the second drug. The treatment schedule with CLFF preceding chemotherapeutic agents showed a similar synergistic growth inhibitory effect to the simultaneous treatment regimen. By contrast, largely antagonistic effects were seen in both two cell lines when cells were treated with the reverse sequence (Figure 3). The results suggested that the simultaneous treatment and administration of CLFF followed by chemotherapeutic agent treatments were better than the reverse sequence treatment.

### 3.3. Apoptosis Effects Mediated by CLFF and Chemotherapeutic Agents

**Figure 4:**
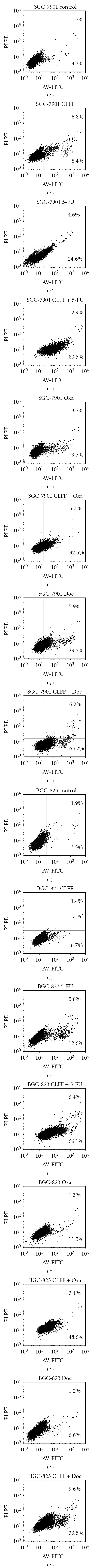
CLFF treatment increase chemotherapeutic agent-induced apoptosis in SGC-7901 (a–h) and BGC-823 (i–p) cells. (a, i) both cell lines were treated without drug. (b, j) both cell lines were treated with CLFF alone. (c, k) both cell lines were treated with 5-fluorouracil alone. (d, l) both cell lines were treated with CLFF combination with 5-fluorouracil. (e, m) both cell lines were treated with oxaliplatin alone. (f, n) both cell lines were treated with CLFF combination with oxaliplatin. (g, o) both cell lines were treated with docetaxel alone. (h, p) both cell lines were treated with CLFF combination with docetaxel. Early apoptotic cells were defined as Annexin-V-positive, PI-negative cells, late apoptotic cells were defined as Annexin-V-positive, PI-positive cells.

To confirm whether CLFF plus chemotherapeutic agents could increase the cell death by inducing apoptosis, flow cytometric analysis was performed to better understand the apoptosis effects of combining CLFF and chemotherapeutic agents. The double staining with both Annexin-V-FITC and PI was employed to distinguish the apoptotic cells from others. SGC-7901 and BGC-823 cell lines were treated with CLFF and anticancer drugs singly and in combination. The doses of agents chosen were close to their respective 50% inhibitory concentrations (IC50). The percentage of the apoptotic cells produced by the individual chemotherapeutic agents was significant increased by the presence of CLFF, indicating that the simultaneous treatment of CLFF and anticancer drugs induced apoptotic in a synergistic manner (Figure 4). Especially, the percentages of the apoptotic cells (early apoptosis plus late apoptosis) induced by CLFF and 5-FU were (8.4 + 6.8), (24.6 + 4.6)% in SGC-7901 cells and (6.7 + 1.4), (12.6 + 3.8)% in BGC-823 cells, while the percentage of them induced by drug combination in both cell lines were increased to (80.5 + 12.9) and (66.1 + 6.4)%, respectively.

### 3.4. CLFF Influence mRNA Expression of Chemotherapeutic Agent Resistance-Related Genes

**Figure 5:**
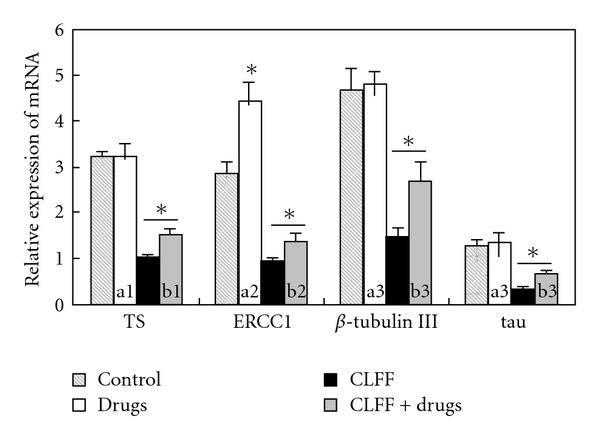
Effect of CLFF on the expression of chemotherapeutic agent resistance-related genes in SGC-7901 cells. The level of ERCC1, TS, *β*-tubulin III and tau mRNA expression, followed treatment with CLFF and every chemotherapeutic agent singly at their respective IC40 or in combination for 24 h, was determined in SGC-7901 cells by real-time RT-PCR. a1: cells were treated with 5-fluorouracil alone. a2: cells were treated with oxaliplatin alone. a3: cells were treated with docetaxel alone. b1: cells were treated with CLFF combination with 5-fluorouracil. b2: cells were treated with CLFF combination with oxaliplatin. b3: cells were treated with CLFF combination with docetaxel. Data point, means of at least three independent experiment; bars, SD. *P < .05 versus control cells.

In an attempt to explain the mechanisms underlying the synergistic interaction between CLFF and chemotherapeutic agents, we hypothesized that CLFF might affect the expression of the investigated chemotherapeutic agent resistance-related genes, i.e. excision repair cross-complementing (ERCC1), thymidylate synthase (TS), class III *β*-tubulin (*β*-tubulin III) and tau, in gastric cancers, influencing sensitivity to those drugs. After incubation with CLFF and every chemotherapeutic agent singly at their respective IC40 or in combination for 24 h, mRNA expressions of these genes in SGC-7901 cells were assessed by quantitative RT-PCR. As shown in Figure 5, the expression levels of ERCC1, TS, *β*-tubulin III and tau were significantly down-regulated after treatment with CLFF alone or CLFF combined with chemotherapeutic agents. However, any of chemotherapeutic agents alone did not down-regulate their respective drug resistance-associated genes, and the expression levels of ERCC1 were even significantly up-regulated by Oxa. Moreover, we also compared the expression levels of chemotherapeutic agent resistance-associated genes between SGC-7901 cells and BGC-823 cells. The results showed that the expression levels of TS, *β*-tubulin III and tau in SGC-7901 cells were lower than those in BGC-823 cells (*P* < .05), while the expression level of ERCC1 was higher than that in BGC-823 cells (*P* < .01).

## 4. Discussion

Active combination chemotherapy has the potential to decrease drug doses, reduce toxicity, and help to overcome the problem of drug resistance. The objective of this report is to investigate whether the antitumor activity of chemotherapeutic agents commonly used in gastric cancer could be enhanced by CLFF, a Chinese herb medicine formula. First, we evaluated the nature of the interaction between CLFF and every chemotherapeutic agent using media-effect analysis. Our findings indicate simultaneous treatment and administration of CLFF followed by chemotherapeutic agent treatments could result in a synergistic interaction between CLFF and series of chemotherapeutic agents for two gastric cancer cell lines. Especially, CLFF had a more intensive synergistic effect on the cytotoxicity of 5-FU. Then we found the combination of CLFF and chemotherapeutic agents could also induce apoptosis in a synergistic manner. At last, we found that the expression of chemotherapeutic agent resistance-related genes was down-regulated by CLFF alone and CLFF combination with chemotherapeutic agents. Our results suggested CLFF appeared a promising candidate for combining with three chemotherapeutic agents. The possible mechanisms might be partially due to the down-regulation of chemotherapeutic agent resistance related genes and the synergistic apoptotic effect.

Herbal medicine formulae as CAM have been accepted by more and more cancer patients in China [8], and its popularity outside China is also undeniable [15]. Many cancer patients had been taking herbal medicine before starting chemotherapy or during chemotherapy. The reasons cited for using CAM in cancer patients included immunomodulation, survival prolongation, quality of life (QOL), reduction of treatment-related toxic effects [5, 6]. Clinical observation found some herbal medicine formulae could improve the outcomes of chemotherapy [16], yet the potential mechanism has not been explained by modern science. Previous studies have demonstrated that many plant-derived compounds have the potential in sensitizing the tumor cells to chemotherapeutic agents or restoring the sensitivity of some drug resistant cells [17]. However, we did not know whether the herbal medicine formulae could sensitize the tumor cells to chemotherapeutic agents. In this study, we observed the interaction between CLFF and chemotherapeutic agents in two gastric cancer cell lines using media-effect analysis. Overall, in low dose inhibition range, CLFF had synergistic effects on the cytotoxicity of all three drugs, suggesting CLFF at least could sensitize the two gastric cancer cell lines to these three chemotherapeutic agents. Similar experiments could be carried out in other cell lines to evaluate further the potential of such combinations against different types of tumor cells.

Currently, it remains unclear to agree with the standard care in gastric cancer treatment. 5-FU, Oxa and Doc represent the most powerful classes of anticancer agents, and triple combinations containing 5-FU has been wide used in gastric cancer management over the past few years [18]. 5-FU-Oxa combinations in numerous phase II studies for advanced gastric cancer have shown response rates and progression free survival (PFS) ranging from 38 to 55% and 5–7 months, respectively [19], and docetaxel has also been shown to be active against advanced gastric cancer as first-line treatment, in combination with 5-FU and cisplatin [20]. 5-FU and Oxa could induce DNA damage directly or indirectly [21, 22], and induced apoptosis predominatly by p53-dependent pathway [23], while Doc induce cell death predominantly via p53-independent pathway [24], such as caspase-dependent pathway. Results of apoptosis analysis here indicated the simultaneous treatment of CLFF and anticancer drugs induced apoptosis in a synergistic manner, which at least in part explain the synergy effects of CLFF in combination of these three drugs. However, the mechanism of CLFF increasing of chemotherapy agents-induced apoptosis need to be further explored by specific studies on apoptosis.

Many experimental studies performed on various cancers have demonstrated that overexpression of chemotherapy agent resistance-related genes is associated with their corresponding drug resistance. TS is the DNA synthesis enzyme that is targeted by 5-FU treatment and plays a very important part in the efficacy of 5-FU. High TS expression level is reported to contribute to a resistance to 5-FU and poor clinical outcome [25, 26]. Cisplatin and other cancer chemotherapeutic agents cause monoadducts and intrastrand or interstrand cross-links in DNA [27]. The removal of adducts from genomic DNA is mainly mediated by the nucleotide excision repair (NER) pathway for the repair of interstrand cross-links [28, 29]. ERCC1 plays a crucial role in NER and has been reported to influence the effectiveness of cisplatin-based therapy for gastric and other cancers in a negative manner [30, 31]. ERCC1 has been shown to be an independent prognostic marker of platinum-based chemotherapy [32]. Of the various microtubule-associated molecules, *β*-tubulin III and tau have been reported to be closely associated with the therapeutic efficacy of taxane-based chemotherapy. Preclinical studies indicate that overexpression of *β*-tubulin III is associated with resistance to tubulin-binding agents [33], and low tau mRNA expression correlates with sensitivity to paclitaxel *in vitro* [34]. Inhibition of tau function could be explored as a potential therapeutic strategy to increase the anticancer activity of paclitaxel [35]. In the present study, we initially found the levels of TS, *β*-tubulin III and tau mRNA in SGC-7901 cells were lower than those in BGC-823 cells, while the levels of ERCC1 mRNA in SGC-7901 cells were higher than that in BGC-823 cells, which might explain why SGC-7901 cells are more sensitive to 5-FU and Doc, while BGC-823 cells are more sensitive to oxaliplatin. We further found that TS, ERCC1, *β*-tubulin III and tau mRNA were significantly down-regulated by CLFF alone or CLFF combination with chemotherapeutic agents, while any of chemotherapeutic agents alone did not down-regulate their respective drug resistance-associated genes, which might explain why CLFF could sensitize 5-FU, Oxa and Doc, and why synergistic effects of simultaneous treatment and administration of CLFF preceding chemotherapeutic agents differ from the reverse schedule. The mechanisms of CLFF influencing these gene expressions remain to be less clear, and the possible mechanisms may be involved in changes of many signal transduction pathways associated with drug–gene interaction, which need further to be studied by analysis of differences in gene expression profiles of different pathways in our future works.

In conclusion, this study has demonstrated that combination of CLFF and a series of chemotherapeutic agents could result in a useful synergistic interaction against gastric cancer, and CLFF has considerable promise as an adjuvant to chemotherapy. Its potential mechanism would be their synergistic effects on apoptosis and the down-regulation of chemotherapeutic agent resistance related genes. Further studies should be studied *in vivo*.

## Funding

Science and Technology Planning Project of Changzhou, Jiangsu Province, China (CS2004214 and C20092025).

## Figures and Tables

**Table 1:** The component of CLFF.

Species (family)	Chinese name	Plant part	Origin
*Rhizoma Paridis*	Chong lou	Stem	Yunnan, China
*Fructus Forsythiae*	Lian qiao	Fruit	Anhui, China
*Radix Codonopsis*	Dang shen	Root	Sichuan, China

**Table 2:** The IC50 doses of CLFF and chemotherapeutic agents.

Cell line	IC50 (mean ± SD)			
	CLFF (mg ml^−1^)	5-Fluorouracil (*μ*m)	Oxaliplatin (*μ*m)	Docetaxel (*μ*m)
SGC-7901	0.22 ± 0.03	42.81 ± 0.03	64.51 ± 4.49	2.74 ± 0.45
BGC-823	0.72 ± 0.06	196.28 ± 33.07	32.41 ± 2.39	17.02 ± 0.91

**Table 3:** Summary of CI values at 20, 50 and 80% fraction affected.

Cell line	Regimen	CI at fraction affected (mean ± SD)			
		20%	30%	50%	80%
SGC-7901	CLFF + 5-FU	0.80 ± 0.18	0.68 ± 0.06	0.71 ± 0.03	0.79 ± 0.02
	CLFF + Oxa	0.87 ± 0.21	0.98 ± 0.15	1.22 ± 0.07	1.80 ± 0.42
	CLFF + Doc	0.68 ± 0.22	0.78 ± 0.17	0.98 ± 0.12	1.55 ± 0.41
BGC-823	CLFF + 5-FU	0.69 ± 0.11	0.74 ± 0.06	0.85 ± 0.02	1.15 ± 0.15
	CLFF + Oxa	0.90 ± 0.19	0.99 ± 0.16	1.21 ± 0.07	1.63 ± 0.18
	CLFF + Doc	0.74 ± 0.23	0.90 ± 0.23	1.24 ± 0.22	2.06 ± 0.15
